# Simultaneous Determination of Bisphenol A and Its Analogues in Food Matrixes: Cumulative Exposure Assessment Following New Regulatory Restrictions—A Systematic Review

**DOI:** 10.3390/foods15061104

**Published:** 2026-03-21

**Authors:** Nika Lovrincevic Pavlovic, Ivan Miskulin, Ivana Kotromanovic Simic, Lea Dumic, Darko Kotromanovic, Maja Miskulin

**Affiliations:** Faculty of Medicine Osijek, Josip Juraj Strossmayer University of Osijek, 31000 Osijek, Croatia; nika.felicita@gmail.com (N.L.P.); ivsimic@mefos.hr (I.K.S.); dumiclea@gmail.com (L.D.); kotromanovic93@gmail.com (D.K.); maja.miskulin@mefos.hr (M.M.)

**Keywords:** bisphenol, analogues, endocrine disruptors, food, analysis, chromatography, LC-MS/MS, dietary exposure, risk assessment, European Union

## Abstract

Recent scientific evidence confirms that there is no safe threshold for bisphenol A intake, prompting strict regulatory actions and new prohibitions in the European Union. As a result, bisphenol A has increasingly been replaced by other analogues that are also toxic but less regulated and insufficiently studied, posing a new risk to human health due to cumulative exposure. Since food is the primary source of exposure to these compounds, this review aimed to evaluate the most appropriate existing chromatographic methods for their determination under newly introduced near-zero tolerance limits, as well as to assess current cumulative dietary exposure and associated health risks. A systematic literature search was conducted in major scientific databases and relevant regulatory sources covering the period from 2015 to 2025, following PRISMA guidelines. Of the 489 identified publications, 22 met the eligibility criteria for full-text analysis. The findings indicate a clear methodological shift towards simultaneous quantification of multiple bisphenol analogues, with LC-MS/MS emerging as the dominant and most robust analytical technique. Dietary exposure to bisphenol A is expected to decline due to stricter regulations; however, this may trigger a rise in the use of its structural analogues as alternatives. Exposure assessments indicate that combined dietary intake of bisphenol A and its analogues can result in a Hazard Index exceeding 1, primarily due to the substantially reduced Tolerable Daily Intake for bisphenol A. This highlights the need for continuous monitoring under stricter regulatory frameworks.

## 1. Introduction

### 1.1. Human Exposure to Bisphenol A and Its Analogues

The widespread application of bisphenol A (BPA), a synthetic compound commonly used as an additive in the production of polycarbonates and epoxy resins for improvement of their physicochemical properties, has led to its pervasive presence in the environment [1,2]. Consequently, BPA has been widely distributed and can be found in numerous consumer goods, including food and beverage containers, internal can linings, kitchenware, bottle caps, surface coatings, toys, medical devices, dental fillings, thermal paper receipts [3], water pipes, and electronic equipment [4]. Bisphenol analogues (BPs), compounds structurally and chemically similar to BPA, are commonly used as alternatives due to the well-documented harmful effects of BPA. Many of these compounds act as endocrine disruptors, just as BPA, and their chemical structures, depending on the attached substituents, significantly influence their physicochemical properties, such as solubility, reactivity, and toxicity. Exposure to these compounds may adversely affect the reproductive and cardiovascular systems, cause metabolic disorders, neurodevelopmental impairments, and organ damage [5]. Over the past decade, BPA has increasingly been replaced worldwide by its analogues in the production of food packaging and other consumer goods [6]. The most commonly used substitutes are BPS, BPF, and BPAF [3,4,7].

While humans are exposed to BPs from a variety of environmental sources due to their widespread presence, diet remains the most significant source of exposure [2,6,8,9,10,11,12]. Therefore, contamination of food and beverages represents one of the most serious food safety issues today [13]. The most significant dietary intake of BPs is attributed to the consumption of canned products [8]. Cans are a practical form of packaging for certain foods due to easier transportation, more efficient cooling compared to glass, greater resistance to damage, and their recyclability [14]. These metal containers are coated with epoxy-based resins that contain BPs. If the chemical reactions involved in the production of these coatings are not fully completed, residual components may be released and subsequently migrate into the food. Since the migration of these compounds is a time-dependent process, the prolonged shelf life of canned foods significantly increases the risk of chemical leaching [15]. Factors that promote this migration include higher temperatures during canning sterilization, contact with acidic, alkaline foods [15,16], and fatty foods. Migration of BPs may take place not only during production or storage, but also during everyday human behaviours and handling [17], such as heating food in plastic containers in the microwave or touching receipts before eating [6].

During typical daily activities, humans are exposed to chemical mixtures rather than single substances, whereby interactions between some compounds may potentially induce synergistic effects [15]. However, the interactions of these chemicals in humans, which may modify the health risks associated with individual pollutants, remain largely unknown [18]. This combined, so-called “cocktail effect” refers to simultaneous exposure to several chemicals, which, even at doses individually considered safe, may collectively produce toxic outcomes. Several studies have highlighted that such combined exposure to endocrine-disrupting compounds may result in long-term adverse effects on human health [13,15]. Therefore, minimizing exposure to these compounds is vital to protect human health from their potentially serious and long-lasting effects [1]. The necessity of monitoring a comprehensive range of BPs is further emphasized by the shift toward cumulative risk assessment in recent global studies. In Europe, Karrer et al. (2019) demonstrated that the combined exposure to BPA and its analogues (BPS, BPF, BPAF) significantly contributes to the overall hazard index (HI), suggesting that single-compound assessments may underestimate public health risks [19]. A study on the Taiwanese population evaluating the cumulative risk of BPA and its substitutes, BPS and BPF, revealed that HI values exceeded 1 in 99% of participants following the implementation of new EFSA regulations, suggesting dietary intake as the primary source of exposure [20]. The shift toward BP substitutes is further highlighted by recent findings in school-aged children from South China [21]. By screening a broad spectrum of analogues, the study confirmed that while BPA remains the primary contaminant, children are simultaneously exposed to various alternatives, including BPS and BPF. Most importantly, under updated safety thresholds, a significant majority of participants exceeded a cumulative HI of 1, underscoring the necessity of a multi-compound approach in risk assessment. Furthermore, very recent evidence from coastal China highlights a critical situation in the seafood chain, where the cumulative hazard quotient (HQ) for BPs was found to exceed safety thresholds (HQ > 1) [22]. All these findings from diverse geographic regions underscore a global shift in the regulatory and scientific landscape, necessitating the development of highly sensitive multi-analyte methods capable of ensuring safety across different international jurisdictions.

### 1.2. Past and Current Regulations on Bisphenols in Food

BPA was approved for use in food packaging in the 1960s [14]. Due to its endocrine-disrupting potential and consequent restrictions in food-contact materials (FCM), its analogues, other BPs, began to be introduced [3,6,12,14,23]. Recent studies have shown that these analogues can also be harmful, in some cases even more than BPA [7,11,14,18]. Canada was the first country to ban BPA in baby bottles in 2010. Next year, the European Union (EU) followed with similar restrictions and banned BPA in plastic infant feeding products, while the Food and Drug Administration (FDA) prohibited BPA in bottles and products intended for children under three years of age in 2012 [7].

Regulatory standards for BPA have tightened significantly over the years. The European Food Safety Authority (EFSA) reduced the Tolerable Daily Intake (TDI) from 50 to 4 µg/kg BW in 2015 [2,7,8,10,11,16,17,18,24], followed by European Chemicals Agency (ECHA) 2017 classification of BPA as a ‘Substance of Very High Concern’ [9], and resulting in the European Commission’s 2018 decision to drastically lower the specific migration limit (SML) from 0.6 mg/kg to 50 µg/kg of food [2,8,9,10,16].

In its scientific opinion published in 2021–2022, EFSA proposed a very low TDI for BPA of 0.04 ng/kg BW, reflecting concerns about immunological effects observed at extremely low exposure levels. In its final opinion adopted in 2023, EFSA revised this value to 0.2 ng/kg BW, representing a 20,000-fold reduction compared with the previous temporary TDI of 4 µg/kg BW established in 2015 [25]. This latest TDI made the establishment of a new SML more challenging. As a result, a completely new regulatory framework has been introduced for BPA and its analogues in the production of materials and articles that may come into contact with food [15,26].

While European Medicines Agency (EMA) and EFSA held conflicting views in 2023, their disagreement revolved around the methodology used for risk assessment. Specifically, EMA was skeptical of using intermediate immunological findings as the primary basis for new rules. However, current EU policy has aligned with EFSA’s assessment, resulting in the recent ban on BPA in FCM [27]. A transitional period is currently in place to give the industry time to adapt. The new rules will be fully enforced starting 20 July 2026 [15].

Similar trends are observed in Asia, particularly in China. While China’s National Health Commission banned BPA in infant feeding bottles in 2011, recent regulatory updates indicate a shift toward more stringent controls for the general population. In July 2025, the China National Center for Food Safety Risk Assessment published a draft amendment of the GB 9685 standard, proposing to lower SML for BPA to 0.05 mg/kg from the current 0.6 mg/kg [28]. This proposed revision aligns China’s SML with the standards contained in the EU Regulation, reflecting a broader movement toward stricter global oversight of BPs. In contrast, the FDA continues to uphold the safety of current BPA exposure levels, despite the growing body of evidence highlighting potential risks that have prompted other global agencies to revise their limits [29]. This highlights the gap between traditional safety views and the move toward more cautious regulations in light of new evidence.

The aim of this systematic review was to evaluate chromatographic techniques for the simultaneous determination of various BPs, following their increased use as structural analogues to replace BPA. Since recent EU regulations have banned these compounds in FCMs, there is an urgent need for highly sensitive analytical methods capable of detecting trace levels. This is critical due to their potent biological activity even at very low concentrations, which poses significant risk to human health. Unlike previous reviews, this study specifically evaluates whether current analytical techniques are sensitive enough to meet the challenges of the 2023 regulatory shifts. By linking methodological performance with the latest cumulative exposure requirements, this review provides a comprehensive overview of current trends and addresses critical gaps in ensuring compliance with near-zero tolerance standards.

## 2. Materials and Methods

### 2.1. Search Strategy

A systematic literature search was conducted across major bibliographic databases: Web of Science (accessed via Clarivate), Scopus (accessed via Elsevier), and Medline (accessed via the Ovid platform) through the time frame of the last 10 years (from 1 January 2015 to 6 November 2025). This timeframe reflects the advancement of analytical methodology prompted by the 2015 EFSA TDI reduction and the subsequent demand for highly sensitive techniques to quantify cumulative BPs exposure under the current stringent near-zero tolerance restrictions. The keywords for major databases included query (bisphenol* OR bp*) AND (determin* OR chromatograph* OR analys* OR quantificat* OR quantit*) AND (food* OR beverage*) AND (dietary AND exposure OR cumulative OR risk AND assessment OR hazard AND index) while for regulation and national agencies keyword were bisphenl OR bisphenol* OR BP* AND food*or just “bisphenol”.

### 2.2. Study Selection and Data Extraction

The PRISMA guidelines were followed throughout the study selection process [30] (see Supplementary Materials, Tables S1 and S2). All retrieved records were first exported to the reference management software Zotero 7.0.32. (*N* = 489). After the removal of 69 duplicate records, 420 unique studies remained that underwent systematic screening by three independent readers based on titles and abstracts of the study to assess their eligibility according to the predefined criteria. The inclusion criteria required the open-access articles discussing toxicological characteristics and health impact of these toxicants, analytical methods for their determination in food samples, as well as the risk assessment of their health impact as demonstrated in Search Strategy. Studies were excluded if they did not address any of these topics.

The first selection was made by sorting the articles according to their titles and grouping the abstracts as follows: articles concerning biomonitoring of bisphenol and its analytes in human samples (64), in vivo experiments (10), determination of bisphenol and analytes in general use items (10), articles dealing with regulations on bisphenols in contact with food (4), articles exclusively related to risk assessment of bisphenols (27), review articles that do not include analytical methods (37), and other articles not exclusively related to the topic of interest (192). Seventy-six articles remained for the second selection, which involved reading the complete articles.

**Figure 1:**
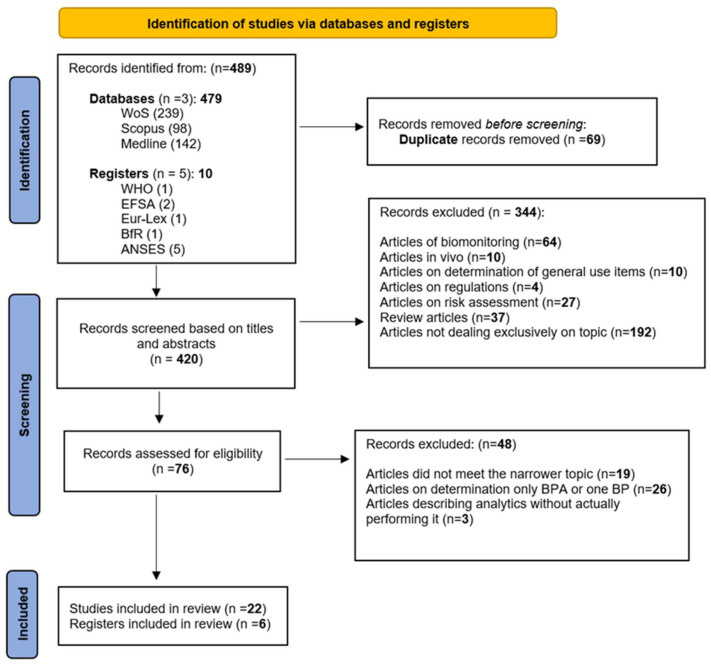
PRISMA flowchart summarizing the screening and exclusion of publications.

Through reading the full articles, out of 76, 19 did not meet the narrower topic, 26 were excluded due to the fact that they mostly determined only one BP or specifically BPA, and 3 described analytical methods but did not actually perform determination in any type of food. Consequently, 22 articles were selected for inclusion, alongside 6 regulatory publications and documents. All record exclusions were performed manually by a human, and no automation tools were used. Figure 1 presents the PRISMA flow diagram illustrating the article selection process for this systematic review.

The 22 selected studies resulted from a rigorous screening process designed to prioritize high-quality research with comprehensive analytical validation. This targeted selection ensures that the findings presented here are based on the most robust and up-to-date evidence currently available. Each study was chosen for its analytical strength in detecting BP mixtures across diverse food matrices.

## 3. Results

### 3.1. Toxicological Characterization and Regulatory Framework of Bisphenols

To better understand the risks associated with BPA substitutes, Table 1 provides a comparative overview of the structural characteristics, toxicological relevance, and current regulatory status of the most prominent BP analogues within the EU. The data highlights how structural similarities to BPA often result in comparable endocrine-disrupting effects, raising concerns regarding the safety of ‘BPA-free’ alternatives. Special emphasis is placed on the toxicological relevance of each compound according to BPA, as well as their inclusion frequency in validated analytical methods found in this systematic review, providing a critical foundation for assessing cumulative exposure.

### 3.2. Methodology Overview and Validation Data

The first part of this systematic review presents a selection of studies focused on the chromatographic determination of BPA and its analogues—or in some cases, solely the analogues—within various food and beverage matrices. The methods in all selected studies had undergone prior validation, with clearly established parameters. Effective chromatographic determination relied primarily on thorough sample preparation, alongside the use of robust and sensitive instrumentation and detectors. The main characteristics and validation parameters essential for detecting low concentrations of BPs in food are summarized in Table 2. Due to inconsistent reporting in the literature, LOD and LOQ concentrations were extracted as provided in the original studies and are generally derived from recovery-based calibrations accounting for the entire analytical procedure.

**Figure 2:**
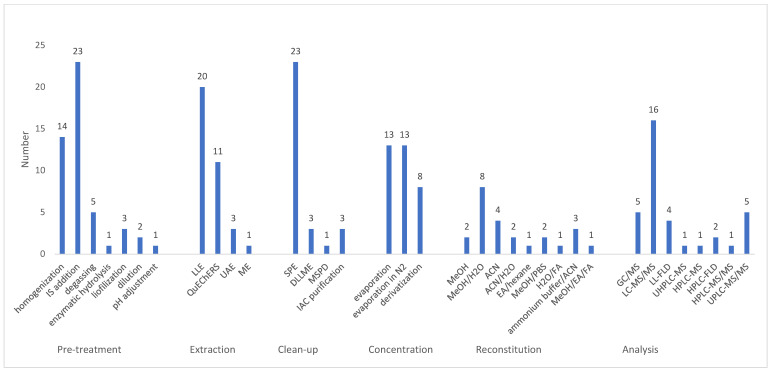
Overview of applied analytical protocols: sample preparation and chromatographic techniques used in selected studies.

To further illustrate the methodological diversity among the selected studies, Figure 2 provides detailed analytical steps employed for BP determination. The graph categorizes the procedures into six key stages: pre-treatment, extraction, clean-up, concentration, reconstitution of the sample, and final analysis. Regarding pre-treatment, the addition of an internal standard (IS) and sample homogenization were the most frequent practices, ensuring higher accuracy (due to lower concentrations) and representativeness. For extraction, Liquid–Liquid Extraction (LLE) and QuEChERS (Quick, Easy, Cheap, Effective, Rugged, and Safe) stood out as the most utilized techniques, while Solid-Phase Extraction (SPE) remained the dominant choice for the clean-up phase. Our findings confirmed a strong preference for liquid chromatography coupled with mass spectrometry (LC-MS), particularly Liquid Chromatography–tandem Mass Spectrometry (LC-MS/MS), which is essential for achieving the high sensitivity required to detect BPs at trace levels in complex food matrices.

For accurate analytical results, the eligible studies included in this review consistently reported and emphasized specific procedures as essential for minimizing background interference and maintaining sample integrity. These procedures include thorough rinsing of all glassware and equipment with methanol (MeOH) prior to use to eliminate potential BP residues, as well as filtration of samples through 0.22 or 0.45 μm polytetrafluoroethylene (PTFE) filters to protect the chromatographic system and ensure analytical purity.

BPs presented in Table 1 were selected based on their frequency of occurrence and prevalence within the literature identified through this systematic review, focusing on those most often mentioned in food studies. Furthermore, toxicological relevance was determined based on their estrogenic activity in laboratory tests and available data on endocrine effects in vivo. Overall toxicity, however, remains highly dependent on exposure route, metabolism, and persistence of each compound [5].

Most of the analyzed studies (Table 2) used standard extraction and detection methods that reach limits of quantification (LOQ) above 1.0 μg/kg. While these results were acceptable under previous regulations, they are no longer sensitive enough to meet the new, much stricter 2023 EFSA standards. These conventional methods are unable to detect BPs at the ultra-trace levels required to confirm that exposure stays below the new TDI. The reason why some studies achieved much lower LOD than others, even when using similar equipment, depends on how the samples were prepared as well as the type of food being tested. The most sensitive methods employed pre-concentration techniques to enhance the enrichment factor, thereby significantly improving the signal-to-noise ratio for instrument detection. Furthermore, derivatization was frequently utilized to modify the BPs, increasing their volatility or detectability and allowing them to be more effectively distinguished from the sample matrix. Finally, it was much easier to reach these ultra-low levels in simple liquids like water or juice than in complex, fatty foods like meat or fish. The complexity of solid food matrices, particularly the high lipid and protein content, poses a major challenge for trace analysis, as these components often interfere with the analyte signal [35].

When linking analytical performance to the 2023 EFSA requirements, it is evident that a significant gap exists. Most current monitoring methods, which typically achieve LOQs in the range of 1.0–10.0 μg/kg, are insufficient for ensuring compliance with the 20,000-fold reduction in TDI. For practical food monitoring, only methods utilizing advanced pre-concentration techniques combined with high-sensitivity LC-MS/MS or GC-MS can currently meet these near-zero tolerance limits. This highlights an urgent need for the standardization of ultra-trace analytical protocols across all food matrices to support the new regulatory framework.

Table 3 provides a quantitative comparison of the data presented in Table 2, highlighting that LC–MS/MS offers the highest analytical sensitivity, with LOQs of 0.00586 μg/kg in some cases, which is essential for meeting the new EFSA standards. In contrast, LC-FLD sensitivity is limited (LOQ ≥ 2.0 μg/kg). Although GC–MS provides adequate LOD, its practical application is hindered by mandatory derivatisation and complex sample preparation, which increase the risk of analyte loss. Consequently, LC–MS/MS remains the most robust tool due to its high selectivity and stable recovery (72.2–121%) across various food matrices, without the need for extensive sample modification. Furthermore, inconsistent reporting of validation parameters across the reviewed studies—including the expression of LOD and LOQ in solution units rather than food matrix concentrations [16], or the omission of LOD with only LOQ reported [11]—reflects differences in established laboratory practices and primary study objectives rather than necessarily indicating methodological deficiency. Nevertheless, such inconsistencies limit the direct comparability of method sensitivity and complicate the evaluation of analytical adequacy against current regulatory requirements. Standardised reporting of these parameters in food matrix units is therefore essential for meaningful cross-study comparisons and for assessing whether existing methods are fit for purpose under the post-2023 regulatory framework.

### 3.3. Cumulative Exposure and Risk Assessment Outcomes

To assess the cumulative risk, the Estimated Daily Intake (EDI) and the Hazard Quotient (HQ) are typically calculated, with the overall mixture risk expressed as the Hazard Index (HI = ∑ HQ) [7,10,11,12,18]. Individual chronic exposure is quantified by determining the *EDI_i_* measured in µg/kg bw/day for each BP and is derived according to the equation:

(1) $${EDI}_{i}=\frac{C_{i}*FR}{BW}$$

where *Ci* is the concentration of specific BP in food (μg/kg), *FR* is the daily food consumption rate (kg/day), and *BW* is body weight (kg). This calculation assumes the application of the necessary unit conversion factor to ensure *EDI_i_* is expressed in μg/kg BW/day. Once the *EDI_i_* is established, the next step is to characterize the individual hazard. To quantify the potential chronic non-carcinogenic hazard posed by each BP, the *HQ_i_* should be calculated. This quotient is defined as the ratio between the *EDI_i_* and the substance’s respective safety benchmark, the *TDI* of BPA, which has recently been set to 0.2 ng/kg BW/day [26,36].

(2) $$HQ_{i}=\frac{{EDI}_{i}}{TDI}$$

Lastly, to determine the overall cumulative hazard of the BP mixture, the HI should be calculated. This methodology employs the dose addition principle and is aligned with the methodological guidelines used by EFSA’s Rapid Assessment of Contaminant Exposure tool (RACE) for chronic non-cancer risk evaluation. It is performed by summing the individual HQ for all identified BPs:

(3) $$HI=\sum HQ=HQ_{BPA}+HQ_{BPF}+HQ_{BPS\ldots }$$

The *HI* serves as a standardized tool for evaluating the cumulative effect resulting from the simultaneous consumption of multiple hazardous compounds. This approach is based on the premise that the intake of a particular food type leads to concurrent exposure to several potentially toxic elements. Within this framework, an HI ≤ 1 indicates that the exposure level is likely without significant risk to human health, whereas an HI > 1 suggests the potential for adverse health effects [18,23].

As noted in some studies, such as Zhou et al. [12], the projected intake likely represents an underestimation of the actual exposure levels, as certain food categories were excluded from the scope of the assessment. Furthermore, even when individual BP levels remain below legal limits, their cumulative impact may exceed the safety threshold for the endocrine system. Given the recent, more stringent regulations, it is evident that current exposure levels still pose a potential risk [15].

## 4. Discussion

### 4.1. Analytical Challenges in Simultaneous Determination

Modern analytical research and increasingly stringent regulatory frameworks demand a fundamental change in food safety monitoring, broadening the scope from BPA to its structural analogues. As the industry, under the pressure of legal restrictions, increasingly uses substitute compounds such as its analogues S, F, or AF and analytical chemistry faces the complex challenge of their simultaneous determination at extremely low levels. An optimized analytical approach is crucial for identifying the ‘cocktail effect,’ where the sum of different analogues in a food product can result in a significant chemical burden that would remain undetected and underestimated if only BPA levels were considered. As shown in Table 2, the majority of the reviewed studies utilize LC-MS/MS. This technique currently represents the ‘gold standard,’ providing the necessary sensitivity that UV (Ultraviolet) detector or FLD (Fluorescence detector) are often unable to achieve. Furthermore, the lowest LOD and LOQ values across the reviewed studies were achieved using LC–MS/MS methods, particularly in beverages and liquid matrices [3,10]. In contrast, methods based on fluorescence detection generally showed the highest LOQ values, especially for BPA and BPAF in canned foods [14,16] highlighting the critical role of detector choice in simultaneous BP analysis.

Sample contamination during analysis, or so-called ‘background contamination,’ also represents a significant invisible challenge. Since BPs are ubiquitous, it is crucial to emphasize the importance of using high-quality glassware, pre-washing and rigorous blanks [12,37]. Furthermore, it should be taken into account that during the simultaneous determination of multiple analogues, the risk of contamination is substantially higher than in the analysis of BPA alone.

Since bisphenol analogues possess different polarities and solubilities, careful optimization of the chromatographic conditions is always essential. Precise parameter adjustment is crucial to achieve the best resolution and sensitivity for all analytes within a reasonable run time. From an analytical perspective, food as a chemically complex matrix remains a challenge for the simultaneous determination of various BPs, as components like lipids in canned meat [7] or edible oils [2] can act as significant analytical interferences that jeopardize accurate results for specific analogues [32]. Due to the extremely low trace concentrations of BPs in food, the use of IS is crucial for accurate quantification and matrix effect compensation, as represented in Table 2 and Figure 2.

Under the new EU Regulation 2024/3190, enforcement of BP restrictions in FCM calls for analytical methods with LOD around 1 μg/kg to reliably demonstrate compliance with non-detectable migration [26]. Many advanced LC–MS/MS methods in our Table 2 achieve LODs far below this benchmark, whereas older methods with higher LOD/LOQ may be insufficient for regulatory surveillance. With the ban on BPA in manufacturing, the LOD serves as the definitive legal benchmark. This “zero tolerance” approach means that any detection renders the product non-compliant, as demonstrable compliance becomes impossible if migration exceeds this analytical threshold.

### 4.2. Cumulative Exposure and Regulatory Shifts

Table 1 clearly demonstrates that ‘BPA-free’ does not necessarily mean safe. Several substitutes, specifically BPB, BPF, and BPS, show hormonal activities very similar to those of BPA, suggesting they may pose comparable risks, while certain analogues, including BPAF, may present even higher risks. The presented data focuses on individual compounds; however, human exposure typically occurs through complex chemical mixtures. This raises concerns about additive toxicity, or even synergy, where the total endocrine effect is greater than that of any single BP alone [38]. In the study by Cunha [7] on canned meat, individual BP levels were moderate, but the total sum reached up to 236 μg/kg. If the authors considered only BPA, they would have missed more than half of the total BP contamination from B, F, AF and Z. An analysis of beverages on the Italian market revealed that relying solely on the measurement of BPA would give a completely misleading picture of product safety. While it was present at very low, almost negligible levels, high concentrations of BPAF and BPM were detected, reaching up to 284 and 1358 μg/kg in beer, respectively. Since BPAF is often more toxic than BPA itself, this example demonstrates that the industry is quietly shifting to analogues that are not yet strictly regulated, making the summation of all present BPs the only way to assess the actual risk to consumers [14]. BPAF represents a unique risk due to its structural properties. Research indicates that its fluorinated substituents fundamentally change how the compound is metabolized, leading to different toxicokinetic profiles compared to BPA [39]. It has been shown to be highly toxic and genotoxic, causing DNA damage even at low exposure levels, and is more harmful than other BP congeners [14].

By analyzing different types of food, Galvez Ontiveros et al. showed the presence of several contaminants, suggesting that different ingredients and complex packaging contribute to the overall exposure. Although BPA was the most frequently detected bisphenol, BPS was the second most common, while BPE was found in only 4.1% of samples. This means that the presence of other analogues further increased the total toxic burden of a meal, confirming that risk cannot be assessed by considering each compound separately, but only by evaluating their combined presence [9]. In fish from the North East Atlantic Ocean, BPs were shown to originate not only from food packaging but also from the ingestion of environmental microplastics. BPA, BPB, and BPE were detected in fish muscle, and their presence was significantly associated with microplastic contamination [18]. These findings again demonstrate that focusing on a single exposure source and a single BP can lead to an underestimation of overall chemical exposure, highlighting the importance of considering the combined presence of BP analogues.

The data summarized in Table 4 call for a critical re-evaluation in light of the recent regulatory shifts initiated by EFSA. The drastic reduction in TDI for BPA has fundamentally altered the interpretation of previous risk assessments. Many studies included in this review, which initially reported HI below the threshold of 1, were categorized as “safe” under the former regulatory framework. However, when these same exposure levels are benchmarked against the new, more stringent TDI, the results frequently exceed safety limits, indicating a significant potential risk to public health. This discrepancy underscores the importance of a dynamic approach to risk assessment. It highlights those concentrations previously considered insignificant now represent a substantial toxicological concern, particularly when accounting for the cumulative effect of multiple BP analogues. Furthermore, it must be emphasized that these projected intakes likely represent an underestimation of the actual exposure, as certain food categories were excluded from the scope of the assessment [12]. The transition from individual to cumulative risk evaluation demonstrates that the current chemical burden in food matrices often surpasses safe levels, demanding more rigorous monitoring and swifter regulatory interventions. However, it is important to acknowledge that the use of the HI model involves certain inherent uncertainties. It assumes that all BP analogues share a common mode of action and that their combined effects are strictly additive, which may not account for potential synergistic or antagonistic interactions. Additionally, due to the lack of specific toxicological reference values for many analogues, the use of BPA’s TDI as a substitute for all BPs is a conservative assumption that may either overestimate or underestimate the actual cumulative risk [20]. Despite these limitations, the HI approach remains the most practical and precautionary tool for screening-level risk assessments. In summary, the evidence from the reviewed studies confirms a measurable shift in food contamination toward BPA analogues that frequently exceed current safety margins. While these findings provide a clear mandate for immediate regulatory action, the persistent migration of these compounds throughout their lifecycle suggests that future monitoring should also incorporate non-target and suspect screening approaches [40].

### 4.3. Limitations and Future Perspectives

Several limitations of this systematic review must be acknowledged. First, comparing BP levels is difficult because studies use different analytical methods with varying sensitivity. Second, the lack of a clear definition for ‘total BPs’ leads to inconsistent results when calculating overall exposure. Additionally, inconsistent reporting of method validation parameters—particularly the absence of LOD and LOQ values expressed in food matrix units across reviewed literature—limits the direct comparability of analytical sensitivity and prevents a definitive assessment of method adequacy against current regulatory thresholds. Furthermore, most of the data comes from only a few regions. This creates a geographic bias, making it hard to apply our findings to the rest of the world. Lastly, many studies were conducted before the new EFSA restrictions, so their risk characterizations may no longer align with current safety levels, calling for a cautious re-interpretation of older data. Calculating the HI as a sum of individual HQ assumes that all BPs contribute equally to the overall risk. However, this approach has a major limitation because reference values for many BPA analogues are still not fully established. Furthermore, different analogues exhibit varying levels of estrogenic potency [38]. Therefore, it is essential to account for these differences in potency and the uncertainty of reference values when interpreting the cumulative risk, rather than relying solely on the additive sum of their calculated quotients. Also, since targeted methods are limited to a specific number of BPs, others may remain overlooked. Shifting from target analysis to suspect screening is necessary to ensure a more accurate risk assessment.

Future research should focus on creating Standard Operating Procedures to ensure that all laboratories monitor the same types of BPs. This would simplify methods for accurately comparing cumulative exposure on a global scale. Since new TDIs are now much lower, it is crucial to develop more sensitive methods capable of detecting even the smallest traces of these compounds. Additionally, instead of only looking for well-known substances, researchers should use advanced scanning techniques to identify new substitutes as soon as they enter the market. Integrating chemical analysis with biological testing will improve our understanding of the combined effects of these mixtures on human health. Furthermore, more studies on duplicate diet studies are needed to account for chemicals that might migrate from kitchenware during cooking. Ultimately, regulations must become proactive rather than reactive. The safety of new BP analogues must be strictly proven before they are widely used as replacements for BPA, preventing the cycle of replacing one harmful chemical with another.

## 5. Conclusions

Although current BP concentrations in food remain detectable, future monitoring must adapt to the drastic reduction in TDI and a fundamental shift from individual to cumulative risk assessment. From a regulatory perspective, it is essential to move beyond ‘BPA-free’ labels and implement ‘total bisphenol’ limits to prevent risks from structural analogues substitution and chemical mixtures. Consequently, analytical efforts must focus on ensuring sufficiently low detection limits—specifically below 1.0 μg/kg—which typically requires the use of high-sensitivity LC-MS/MS or GC-MS systems, as less sensitive techniques are no longer adequate for demonstrating compliance with 2023 EFSA standards. In the context of food safety, well-defined quantification limits are not a mere formal requirement—they directly determine whether a method is capable of detecting contamination at toxicologically relevant concentrations. Without this information, meaningful comparison across studies and against current regulatory thresholds becomes inherently limited. This is especially concerning under the current near-zero tolerance framework, where the sensitivity of the analytical method is itself the limiting factor in ensuring consumer protection. Future studies should therefore prioritize transparent and standardized reporting of validation parameters in food matrix units to enable reliable, cross-study assessments of analytical adequacy. The observed variability in such reporting—reflecting differences in established laboratory practices and primary study objectives—underscores the urgent need for harmonized standards that explicitly link method performance to current regulatory benchmarks. Finally, advancing toward comprehensive screening methodologies is vital for the early detection of novel BP substitutes, enabling food safety authorities to ensure robust preventive monitoring and mitigate the accumulation of unregulated analogues throughout the food supply chain.

## Figures and Tables

**Table 1:** A comparative overview of the structural, toxicological, and legal status of bisphenol analogues in the EU.

Phenol Acronym	Structure	Molecular Formula	Occurrence in Included Studies (%)	Toxicological Relevance Compared to BPA	EU Regulatory Status (FCM)
BPA	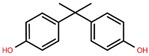	C_15_H_16_O	96.1	Reference compound (baseline endocrine activity)	Not authorized
BPB	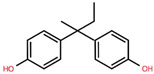	C_16_H_18_O_2_	73.1	Higher estrogenic potency in vitro; increased lipophilicity may enhance bioaccumulation	Not authorized; SVHC (REACH)
BPE	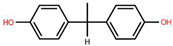	C_14_H_14_O_2_	46.2	Lower or comparable estrogenic activity; limited toxicological data	Not authorized; group restriction *
BPF	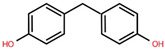	C_13_H_12_O_2_	84.6	Comparable estrogenic and endocrine-disrupting activity in vitro and in vivo	Not authorized; group restriction *
BPS	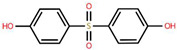	C_12_H_10_O_4_S	65.4	Comparable endocrine-disrupting effects despite lower estrogen receptor affinity; higher environmental persistence	Not authorized; group restriction *
BPP	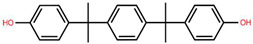	C_24_H_26_O_2_	26.9	Higher estrogen receptor binding affinity; limited toxicological data	Not authorized; group restriction *
BPZ	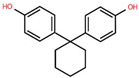	C_18_H_20_O_2_	30.8	Limited toxicological data	Not authorized; group restriction *
BPAF	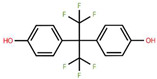	C_15_H_10_F_6_O_2_	42.3	Significantly higher estrogenic and antiandrogenic activity than BPA	Not authorized; SVHC (REACH)
BPAP	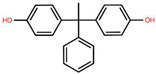	C_20_H_18_O_2_	26.9	Potentially higher endocrine activity; limited experimental data	Not authorized; group restriction *

Data based on references [5,18,24,25,27]. Chemical structures were drawn using Ketcher v2.25.0.; * Refers to the general chemical structures defined in Annex I covering all BPs [26].

**Table 2:** Characteristics of Sample Matrix, Extraction Procedures, and Method Validation Data from selected studies (22).

Ref.	Matrix		Sample Preparation and Chromatography	BP	Recovery (%)	LOQ (μg/kg)	LOD (μg/kg)
[18]	Fish tissue from NE Atlantic Ocean	Muscle	Pre-treatment: lyophilization; IS added Extraction: QuEChERS (ACN + salts; salting-out) Clean-up/concentration/derivatization: DLLME GC–MS	BPA	78–106	1.3	0.9
				BPB	72–75	1.3	0.9
				BPE	81–89	2.2	1.5
				BPF	82–90	2.6	1.9
				BPZ	63–72	2.4	1.65
				BPAF	93–118	2.6	1.7
				BPAP	66–73	3.0	2.4
		Liver	Pre-treatment: lyophilization; IS added Extraction: QuEChERS (ACN + salts; salting-out) Clean-up: d-SPE Concentration/derivatization: DLLME GC–MS	BPA	78–104	3.6	1.8
				BPB	76–97	3.6	2.2
				BPE	70–78	7.7	5.1
				BPF	70–77	7.3	5.5
				BPZ	87–104	7.7	5.5
				BPAF	68–105	7.3	5.8
				BPAP	92–105	7.7	5.5
[24]	Canned foods (104)	Solid samples (Meat, fish, corn & beans, fruit, vegetables, sauces)	Pre-treatment: homogenization; IS added Extraction: LLE (ACN + HEX) sonication/shaking/centrifugation Clean-up: HEX wash; filtration Concentration: N_2_ evaporation; MeOH/H_2_O reconstitution LC–MS/MS	BPA	92.13–106.50	4.27–15.65	1.41–5.16
				BPF	84.76–106.38	3.43–9.28	1.13–3.06
				BPS	81.06–113.60	0.44–2.92	0.14–0.96
		Liquid samples (alcoholic & non-alcoholic beverages, coffee)	Pre-treatment: IS added Extraction: LLE (ACN/water/HEX + NaCl; salting out) sonication/shaking/centrifugation Clean-up: optional SPE (C18) Concentration: N_2_ evaporation; MeOH/H_2_O reconstitution LC–MS/MS	BPA	97.17–106.19	5.17–10.95	1.71–3.61
				BPF	96.10–106.10	0.96–7.34	0.32–2.42
				BPS	93.28–105.60	0.47–5.67	0.15–1.87
[7]	Canned food (30)	Meat (pâtés, sausages, meal foods)	Pre-treatment: homogenization; IS added Extraction: LLE (heptane/water) + QuEChERS (ACN + salts; salting out) Clean-up: d-SPE (Z-Sep + C18); DLLME-AD Concentration: — Final prep: GC–MS	BPA	75–95	0.5	0.15
				BPAF	79–85	1.5	0.4
				BPAP	72–101	2.0	0.7
				BPE	66–70	5.0	2.0
				BPF	67–73	1.5	0.4
				BPP	72–90	2.5	1.5
				BPS	78–98	1.5	0.4
				BPZ	78–80	2.5	1.5
				BPB	68–97	1.0	0.3
[17]	Canned tuna in oil or aqua medium (33)	Tuna	Pre-treatment: homogenization Extraction: LLE (HEX/ACN); ultrasound; stirring; centrifugation Clean-up: filtration Concentration: — LC–FLD	BPA	91–100	4.33	1.3
				BPB	94–105	10.0	3.0
		Oil from tuna	Pre-treatment: IS added Extraction: LLE HEX Clean-up: Florisil SPE Concentration: evaporation; ACN reconstitution LC–FLD	BPA	77–100	3.7	1.1
				BPB	72–99	3.0	0.9
		Aqua for tuna	Pre-treatment: centrifugation Extraction: centrifugation, filtration Clean-up: — Concentration: — LC–FLD	BPA	89–102	3.9	1.1
				BPB	87–100	3.5	1.0
[23]	Milk	Raw and processed	Pre-treatment: IS added; enzymatic hydrolysis (total BPA and derivates) Extraction: QuEChERS (ACN + salts; salting out) Clean-up: d-SPE Concentration: — LC–MS/MS	BPA	98.7–102.4	0.64	0.19
				BPS	80.1–89.6	0.03	0.01
				BPF	96.4–102.5	0.67	0.20
				BPAF	94.9–100.0	0.10	0.03
				BPB	99.9–105.8	0.22	0.06
				BPE	97.2–105.4	0.73	0.09
[14]	Canned beverages	Beer & Energy drinks	Pre-treatment: degassing Extraction: LLE (MeOH) Clean-up: SPE (MeOH; H_2_O/MeOH) Concentration: N_2_ evaporation-dissolve in ACN LC–FLD	BPA	92.6–100.3	9.48	2.85
				BPF	99.5–106.7	9.27	2.78
				BPE	86.2–106.6	8.25	2.47
				BPB	94.6–105.2	6.87	2.06
				BPAF	78.6–96.0	12.09	3.63
				BPM	85.3–89.1	8.63	2.5
[6]	DDSF (solid food)		Pre-treatment: homogenization; IS added Extraction: modified QuEChERS (ACN + salts; salting out) Clean-up: d-SPE (GCB) Concentration: evaporation; ACN reconstitution; derivatization GC–MS	BPB	93.9–113	0.3	n.r.
				BPF		0.3	n.r.
				BPP		0.3	n.r.
				BPS		0.3	n.r.
				BPZ		0.5	n.r.
[9]	Solid foods and dietary samples	Unprocessed, minimally processed, processed and ultra-processed foods	Pre-treatment: lyophilization; IS added Extraction: QuEChERS (ACN + salts; sonication) Clean-up: d-SPE (MgSO_4_ + PSA) Concentration: evaporation; MeOH/H_2_O reconstitution UHPLC–MS	BPA	94–104	0.9	0.3
				BPF	94–104	0.5	0.1
				BPS	91–103	1	0.3
				BPAF	95–103	0.4	0.1
				BPP	92–104	4	1
				BPE	93–105	1	0.3
				BPB	93–104	0.9	0.3
[31]	Mussels	Raw, steamed, canned	Pre-treatment: draining; homogenization; IS added Extraction: MSPD (Florisil/Na_2_SO_4_ + ACN elution) Clean-up: intrinsic to MSPD Concentration: N_2_ evaporation; MeOH/H_2_O reconstitution HPLC–MS	BPA	73.6–77.3	2.28–2.33	0.68–0.70
				BPF	80.5–82.7	0.44–1.77	0.13–0.53
				BPS	79.9–82.5	0.21–1.57	0.06–0.47
[8]	Canned seafood	Spicy clam, Antarctic krill, spicy shrimps, spicy scallop, smoked mussel, oysters in oil	Pre-treatment: homogenization (ice bath) Extraction: MAE (HEX/AC) + LLE (ACN) Clean-up: SPE Concentration: rotary evaporation; MeOH/H_2_O reconstitution HPLC–FLD	BPA	88.85–92.08	2.73	0.84
				BPF	91.18–101.6	2.00	0.59
[15]	Ready-to-eat fish & meat foods (120)	Fish (tuna, mackerel, sardines, salmon, crab, anchovies, clams, shrimp, cuttlefish, and lumpfish) Meat (chicken, beef, pork)	Pre-treatment: sample + ACN Extraction: QuEChERS (salts; salting out) Clean-up: d-SPE (MgSO_4_, PSA, C18) Concentration: — HPLC–MS/MS	BPA	n.r.	1.3	0.4
				BPS		1.7	0.5
				BPF		5.0	1.5
				BPB		1.7	0.5
				BPE		10.0	3.0
				BPZ		1.7	0.5
				BPP		5.0	1.5
				BPAP		5.0	1.5
				BPAF		3.3	1.0
[16]	Canned foods (16)	Fish (tuna, sardines), seafood (clams, mussels), vegetables (olives, asparagus, tomato), grains (sweet corn) and fruit (peach in syrup).	Pre-treatment: separation liquid from solid; homogenization Extraction: LLE (HEP) + LLE (ACN/H_2_O) Clean-up: filtration Concentration: — HPLC–FLD; LC–MS/MS for confirmation	BPA	71–107	12.5	5.0
				BPB	72–106	12.5	5.0
				BPC	80–105	12.5	5.0
				BPE	70–82	12.5	5.0
				BPF	74–114	12.5	5.0
				BPG	70–85	12.5	5.0
[4]	Canned food and beverages (22)	Canned pineapple, peaches, ravioli, farce vol-au-vent, soup, fruit puree, tuna cola light, lemon, beer	Pre-treatment: separation; homogenization; IS added Extraction: LLE (water) + QuEChERS (ACN + salts; salting-out) Clean-up: d-SPE Concentration: N_2_ evaporation; derivatization (BSTFA) GC–MS	BPA	60–115 (Mean recovery–acceptable range; except tuna: 25–46% for BPAP; BPM; BPP; BPBP; BPPH)	1.71	0.51
				BPAF		1.32	0.39
				BPAP		1.96	0.59
				BPB		0.33	0.10
				BPBP		0.10	0.03
				BPC		2.16	0.65
				BPE		0.84	0.25
				BPF		0.15	0.04
				BPG		1.23	0.37
				BPM		1.65	0.5
				BPPH		0.37	0.11
				BPP		0.15	0.04
				BPS		5.55	1.66
				BPTMC		1.93	0.58
				BPZ		0.67	0.20
				TMBPF		0.92	0.27
[3]	Beverages	Alcoholic (whiskey, blonde beer, red wine) and non-alcoholic drinks (cola soft drink, mineral water, English breakfast tea)	Carbonated beverages Pre-treatment: degassing IS added Extraction: SPE (MeOH → H_2_O/FA) Clean-up: SPE Concentration: evaporation; reconstitution (ACN) derivatization LC–MS/MS Non-alcoholic beverages: Pre-treatment: IS added Extraction: SPE (H_2_O/FA → MeOH elution) (Alcoholic drinks diluted with water/FA—SPE extraction) Clean-up: SPE Concentration: evaporation; reconstitution (MeOH/EA/FA) derivatization LC–MS/MS Juices with pulp: Pre-treatment: IS added Extraction: LLE (ACN/MeOH) + UAE Clean-up: SPE (same conditions as above) Concentration: evaporation; reconstitution water/FA derivatization LC–MS/MS	BPA	n.r.	0.0061–0.0235	0.0018–0.0071
				BPF		0.008–0.0272	0.0024–0.0082
				BPE		0.0052–0.0057	0.0016–0.0017
				BPB		0.0099–0.0116	0.003–0.0035
				BPZ		0.0088–0.009	0.0027
				BPAF		0.0053–0.0068	0.0016–0.002
				BPAP		0.0078–0.0079	0.0023–0.0024
				BPP		0.0063–0.0067	0.0019–0.002
				BPS		0.00586–0.0646	0.0176–0.0194
[32]	Ready-to-eat meals	Meat or fish portion of main course + starchy component (rice/potato/pasta) a vegetable portion and/or a sauce	Pre-treatment: homogenization (sand/Na_2_SO_4_); IS added Extraction: UAE (ACN/MeOH) Clean-up: SPE (PSA) Concentration: evaporation; reconstitution (EA/HEX); derivatization LC–MS/MS	BPA	79–101	0.243	0.073
				BPAF		0.066	0.025
				BPAP		0.039	0.031
				BPF		0.092–0.199	0.043–0.110
				BPP		0.072	0.031
				BPS		0.334	0.140
				BPZ		0.038	0.038
				BPB		0.081	0.033
				BPE		0.053	0.031
[33]	Coffee	Capsule & French press	Pre-treatment: IS spiked Extraction: LLE (EA) Clean-up: — Concentration: reconstitution (MeOH) UPLC–MS/MS	BPA	81.5	1.1	0.34
				BPF	90.6	1.1	0.35
				BPS	100.0	0.7	0.23
[13]	Plant-based Beverages	Soya, oats, rice almond, coconut beverage	Pre-treatment: homogenization Extraction: LLE (MeOH) Clean-up: SPE under vacuum Concentration: rotary evaporation; reconstitution (H_2_O/MeOH) LC–MS/MS	BPA	98–105	0.78	0.24
				BPB	98–101.3	0.78	0.24
				BPS	78.0–85.3	0.78	0.24
[10]	Food samples	Seven groups as: beverages (soft drinks, fruit juice, tea drinks) seafood (fish, shrimp, squid), milk products (milk, yogurt, cheese), vegetables (pepper, tomato, corn, mushroom, olive, cucumber, peas), fats and oils (olive, sesame, coconut and corn oils), condiments (soy sauce, ketchup, mayonnaise, mustard paste, chili powder) others (soup, jelly, jam, pancake syrup, custard powder)	Solid samples: Extraction: LLE (EA) Clean-up: SPE (HLB) Concentration: N_2_ evaporation; reconstitution (ACN/water) LC–MS/MS Beverages: Pre-treatment: degassing (if carbonated); IS added Extraction: LLE (EA) with rotary shaking Clean-up: SPE (HLB) Concentration: evaporation; reconstitution in ACN/water LC–MS/MS Dairy products: Pre-treatment: IS added Extraction: LLE (ACN) Clean-up: SPE (HLB), after dilution with FA Concentration: evaporation; ACN/water reconstitution LC–MS/MS	BPA	80.5–103.5	0.01	0.003–0.006
				BPF	81.4–103.5	0.05	0.015–0.03
				BPS	81.2–103.8	0.02	0.006–0.010
[2]	Edible liquids	Water, whiskey, fermented drinks (palm wine and sorghum beverages), crude palm oil	Pre-treatment: IS added; degassing (when needed) Extraction: Water/beverages: LLE (EA) Oils: SPE (HEX + MeOH/H_2_O) Clean-up: SPE Concentration: evaporation; MeOH/H_2_O reconstitution UPLC–MS/MS	BPA	83.7–121.0	0.09–0.56	0.03–0.17
				BPB	72.2–115.8	0.04–0.2	0.01–0.06
				BPF	79.9–119.5	0.01–0.20	0.03
[11]	Chinese total diets	Various food	Plant-based foods: Pre-treatment: homogenization; IS added Extraction: LLE (ACN) Clean-up: IAC purification Concentration: N_2_ evaporation; reconstitution (MeOH/PBS) UHPLC–MS/MS Animal-derived foods: Pre-treatment: homogenization; IS added Extraction: LLE (ACN) Clean-up: IAC Concentration: N_2_ evaporation; reconstitution (MeOH/PBS) UHPLC–MS/MS Beverages: Pre-treatment: degassing; dilution; pH adjustment to 8.5 Extraction: LLE (direct to IAC) Clean-up: IAC Concentration: N_2_ evaporation; MeOH/water reconstitution UHPLC–MS/MS	BPA	87.2–116.3	0.1–0.5	n.r.
				BPF	86.4–113.3	0.2–1.0	n.r.
				BPS	84.6–116.8	0.013–0.05	n.r.
				BPAF	87.7–117.5	0.013–0.05	n.r.
				BPB	87.3–116.7	0.1–0.5	n.r.
[34]	Different foods (79)	Convenience foods Canned vegetable oils Olives Soft drinks	Pre-treatment: homogenization Extraction: LLE (ACN) Clean-up: SPE Concentration: N_2_ evaporation; MeOH/H_2_O reconstitution LC–MS/MS	BPA	86–97	3.0	1.0
				BPE	86–104	4.0	2.0
				BPS	90–105	4.0	2.0
				BPF	86–93	3.0	1.0
				BPB	86–99	3.0	1.0
[12]	Food (349)	12 categories: Water, beverages, rice, wheat flour, shellfish, fish, fresh meat, vegetables, canned cereal, canned fish, canned meat and others: edible oil, egg, honey	Sample preparation divided in 3: Water: Pre-treatment: IS added; NH_3_ addition Extraction: QuEChERS (ACN + FA + salts; salting-out) Clean-up: Oasis MAX SPE Concentration: N_2_ evaporation; reconstitution (ammonium buffer/ACN) LC–MS/MS Beverages: Pre-treatment: IS added Extraction: QuEChERS (ACN + FA; salting-out) Clean-up: Oasis MAX SPE Concentration: N_2_ evaporation; ammonium buffer/ACN reconstitution LC–MS/MS Others: Pre-treatment: homogenization; IS added Extraction: QuEChERS (water + ACN + FA; salting out) Clean-up: Oasis MAX SPE Concentration: N_2_ evaporation; reconstitution (ammonium buffer/ACN) LC–MS/MS	BPA	87–116	0.014–0.120 (aq) 0.1–4.0 (other)	0.004–0.055 (aq) 0.05–1.2 (other)
				BPS	87–112		
				BPB	76–112		
				BPF	81–110		

n.r.—not reported; For liquid samples, concentrations are expressed as µg/kg assuming a density of 1 kg/L. For studies reporting LOD and LOQ exclusively as instrumental limits in solution units, direct conversion to food matrix concentrations (μg/kg) was not feasible without explicit sample weight-to-volume data. Specifically, Lestido-Cardama et al. [16] expressed LOD and LOQ as 0.005 mg/L and 0.0125 mg/L respectively, determined from the instrumental calibration curve; these values are presented in the table as 5.0 and 12.5 μg/kg based on ppb unit equivalence, applicable uniformly to all analytes as the authors reported identical limits across all compounds. Morgan et al. [6] reported a Method Quantitation Limit (MQL) of 0.3 ng/g, functionally equivalent to LOQ, with no corresponding LOD reported. Yao et al. [11] reported LOQ values only, with no LOD provided. The absence of LOD and/or LOQ values expressed in food matrix units in some studies limits direct cross-study comparison of method sensitivity, particularly in the context of current EFSA near-zero tolerance requirements.

**Table 3:** Comparison of LOD, LOQ, and Recovery across methods from Table 2.

Analytical Method	LOD Range (μg/kg)	LOQ Range (μg/kg)	Recovery (%)
LC-FLD	0.9–3.63	2.0–12.09	68.0–106.7
GC-MS	0.03–5.8	0.1–7.7	60.0–118.0
LC-MS/MS	0.0016–5.16	0.00586–15.65	72.2–121.0

**Table 4:** Estimated Daily Intake (EDI) and Hazard Index (HI) comparison for BPs across selected studies, based on former and current EFSA regulatory thresholds.

Ref	State	Year	Sample	Bisphenols Analyzed	EDI (μg/kg bw/day)	Former HI *	HI Current **
[18]	Portugal	2020	Fresh fish	BPA, BPB, BPE, BPF, BPZ, BPAF, BPAP	0.0084	<1	**>1**
[24]	Korea	2018	Canned foods	BPA, BPF, BPS	0.02216	<1	**>1**
[7]	Portugal	2020	Canned meat	BPA, BPAF, BPAP, BPE, BPF, BPP, BPS, BPZ, BPB	33.47	**>1**	**>1**
[17]	Italia	2015	Canned tuna	BPA, BPB	0.007	<1	**>1**
[16]	Spain	2021	Canned food & beverages	BPA, BPB, BPC, BPE, BPF, BPG	0.018–0.21	<1	**>1**
[15]	Italia	2025	Ready-to-eat-meat and fish foods	BPA, BPS, BPF, BPB, BPE, BPZ, BPP, BPAP, BPAF	0.88–16.66	-	**>1**
[8]	China	2022	Canned seafood	BPA, BPF	0.0129	<1	**>1**
[10]	Saudi Arabia	2022	Various food (7 groups)	BPA, BPF, BPS	0.2867–0.3078	**>1**	**>1**
[2]	Africa	2023	Sachet water	BPA, BPB, BPF	1.67	<1	**>1**
[11]	China	2020	Various food (total diet study)	BPA, BPB, BPF, BPAF, BPS	0.222–0.508	<1	**>1**
[12]	China	2019	Various food (12 categories)	BPA, BPS, BPF, BPB	0.056–0.0761	<1	**>1**

* 4 µg/kg bw/day; ** 0.2 ng/kg bw/day (2023).

## Data Availability

No new data were created or analyzed in this study. Data sharing is not applicable to this article.

## Supplementary Materials

The following supporting information can be downloaded at: https://www.mdpi.com/article/10.3390/foods15061104/s1, Table S1: PRISMA 2020 for Abstract Checklist; Table S2: PRISMA 2020 Checklist.

## Abbreviations

AA	Acetic Anhydride
AC	Acetone
ACN	Acetonitrile
AD	Absorbed Dose
BPA	Bisphenol A
BPAF	Bisphenol AF
BPB	Bisphenol B
BPE	Bisphenol E
BPF	Bisphenol F
BPS	Bisphenol S
BSTFA	N,O-Bis(trimethylsilyl)trifluoroacetamide (derivatization agent)
BW	Body Weight
DLLME	Dispersive Liquid–Liquid Microextraction
DNA	Deoxyribonucleic Acid
EA	Ethyl Acetate
EC	European Commission
ECHA	European Chemicals Agency
EFSA	European Food Safety Authority
EMA	European Medicines Agency
EU	European Union
FA	Formic Acid
FCMs	Food Contact Materials
FDA	Food and Drug Administration
FLD	Fluorescence Detector
FR	Daily Food Consumption Rate
GC-MS	Gas Chromatography–Mass Spectrometry
GLP	Good Laboratory Practice
HDI	Human Daily Intake
HEP	Heptane
HEX	Hexane
HI	Hazard Index
HLB	Hydrophilic-Lipophilic Balance (sorbent)
HPLC	High Performance Liquid Chromatography
HQ	Hazard Quotient
IAC	Immunoaffinity Chromatography
IS	Internal Standard
LC-MS/MS	Liquid Chromatography–tandem Mass Spectrometry
LLE	Liquid–Liquid Extraction
LOD	Limit of Detection
LOQ	Limit of Quantitation
MAE	Microwave extraction
MeOH	Methanol
NE	North East
PBS	Phosphate-Buffered Saline
PSA	Primary Secondary Amine (sorbent)
PTFE	Polytetrafluoroethylene (Teflon)
QuEChERS	Quick, Easy, Cheap, Effective, Rugged, and Safe
RACE	Risk Assessment of Chemical Exposure
REACH	Registration, Evaluation, Authorization and restriction of Chemicals
SML	Specific Migration Limit
SPE	Solid Phase Extraction
SVHC	Substance of Very High Concern
TDI	Tolerable Daily Intake
UAE	Ultrasound-Assisted Extraction
UHPLC	Ultra-High Performance Liquid Chromatography
