# Supplementary material for: Simultaneous Determination of Bisphenol A and Its Analogues in Food Matrixes: Cumulative Exposure Assessment Following New Regulatory Restrictions—A Systematic Review
Source: Foods. 2026 Mar 21;15(6):1104. doi: 10.3390/foods15061104 (PMC13025183; doi:10.3390/foods15061104)
Supplement: Supplementary file 1 [file foods-15-01104-s001.zip › foods-4157738-Table S2.pdf]

PRISMA 2020 Checklist

Section and Topic	Item #	Checklist item	Location where item is reported
**TITLE**			
Title	1	Identify the report as a systematic review.	Title, lines 1-4; Page 1
**ABSTRACT**			
Abstract	2	See the PRISMA 2020 for Abstracts checklist.	In accordance with the PRISMA 2020 checklist<br>Line 11-28, page 1
**INTRODUCTION**			
Rationale	3	Describe the rationale for the review in the context of existing knowledge.	The introduction discusses the dangers of BPA and its analogues in food contact products. More stringent regulations bring new obstacles that this review wants to highlight, both in the detection of these chemicals and its exposure to human health; lines 38-103 (exposure) page 2-3; lines 105-147; pages 3-4 (regulations)
Objectives	4	Provide an explicit statement of the objective(s) or question(s) the review addresses.	Clearly presented in the end of the introduction: lines 149-159, page 4
**METHODS**			
Eligibility criteria	5	Specify the inclusion and exclusion criteria for the review and how studies were grouped for the syntheses.	Inclusion and exclusion criteria described: Lines 181-185, Page 4; study selection was explained in lines 175-200, Page 4-5
Information sources	6	Specify all databases, registers, websites, organisations, reference lists and other sources searched or consulted to identify studies. Specify the date when each source was last searched or consulted.	Search strategy (databases, registers, timeline) was explained in detail in lines 162-173, Page 4, and PRISMA flowchart in Figure 1 (page 5)
Search strategy	7	Present the full search strategies for all databases, registers and websites, including any filters and limits used.	Keywords, Boolean operators and databases, time limits are reported in lines 169-173, Page 4
Selection process	8	Specify the methods used to decide whether a study met the inclusion criteria of the review, including how many reviewers screened each record and each report retrieved, whether they worked independently, and if applicable, details of automation tools used in the process.	Screening was done by three independent readers (line 180, page 4) and all record exclusions were performed manually by a human, with no automation tools (lines 198-199, page 5)
Data collection process	9	Specify the methods used to collect data from reports, including how many reviewers collected data from each report, whether they worked independently, any processes for obtaining or confirming data from study investigators, and if applicable, details of automation tools used in the process.	Data collection process: Data regarding analytical techniques, BP levels, and exposure metrics were extracted from each study.
Data items	10a	List and define all outcomes for which data were sought. Specify whether all results that were compatible with each outcome domain in each study were sought (e.g. for all measures, time points, analyses), and if not, the methods used to decide which results to collect.	The primary outcomes were to define highly sensitive chromatography methods for quantifying BPs in food and their cumulative exposure (lines 149-159, page 4)
	10b	List and define all other variables for which data were sought (e.g. participant and intervention characteristics, funding sources). Describe any assumptions made about any missing or unclear information.	Other variables extracted included BP types (BPA and analogues), food matrices (Table 2) and calculated hazard indexes where applicable (Table 4)
Study risk of bias assessment	11	Specify the methods used to assess risk of bias in the included studies, including details of the tool(s) used, how many reviewers assessed each study and whether they worked independently, and if applicable, details of automation tools used in the process.	The risk of bias in the included studies was not formally assessed using a specific tool, as the review primarily focused on analytical methodology and descriptive exposure data
Effect measures	12	Specify for each outcome the effect measure(s) (e.g. risk ratio, mean	The synthesis focused on comparing most frequent BPs in food (Table 1),

Section and Topic	Item #	Checklist item	Location where item is reported
		difference) used in the synthesis or presentation of results.	analytical performance parameters (e.g., LOD and LOQ and recovery) – Table 2 and comparison of their LODs and LOQs (Table 3), and Hazard Index (HI) values for risk assessment (Tables 4)
Synthesis methods	13a	Describe the processes used to decide which studies were eligible for each synthesis (e.g. tabulating the study intervention characteristics and comparing against the planned groups for each synthesis (item #5)).	Studies were grouped for synthesis based on whether they focused on analytical methodology (Table 2) or exposure/health risk data (Table 4)
	13b	Describe any methods required to prepare the data for presentation or synthesis, such as handling of missing summary statistics, or data conversions.	Results were synthesized qualitatively and presented in structured tables to compare food matrix, extraction procedures, validation data (Table 2) and cumulative exposure risks according to new EU Regulation (Table 4)
	13c	Describe any methods used to tabulate or visually display results of individual studies and syntheses.	To verify the sensitivity of BPs detection against new low TDI limits, the LOD, LOQ and expressed recovery are emphasized in the table.
	13d	Describe any methods used to synthesize results and provide a rationale for the choice(s). If meta-analysis was performed, describe the model(s), method(s) to identify the presence and extent of statistical heterogeneity, and software package(s) used.	NA (No meta-analysis conducted)
	13e	Describe any methods used to explore possible causes of heterogeneity among study results (e.g. subgroup analysis, meta-regression).	NA (No meta-analysis conducted)
	13f	Describe any sensitivity analyses conducted to assess robustness of the synthesized results.	The chromatographic methods in all selected studies were validated and only studies detecting more than one BP were included (lines 225-228)
Reporting bias assessment	14	Describe any methods used to assess risk of bias due to missing results in a synthesis (arising from reporting biases).	Reporting bias was addressed by performing a comprehensive search across multiple databases and regulatory sources to ensure all relevant published data were captured
Certainty assessment	15	Describe any methods used to assess certainty (or confidence) in the body of evidence for an outcome.	Formal certainty assessment was not performed due to the descriptive nature of the synthesis of analytical methods.
**RESULTS**			
Study selection	16a	Describe the results of the search and selection process, from the number of records identified in the search to the number of studies included in the review, ideally using a flow diagram.	Results of the selection process were presented using a PRISMA flow diagram (Figure 1, page 5)
	16b	Cite studies that might appear to meet the inclusion criteria, but which were excluded, and explain why they were excluded.	All inclusion and exclusion studies are explained in detail in section in Study selection and Data Extraction (2.2. section), as well as in PRISMA flowchart (page 5) - Studies that focused on non-food exposure or lacked specific chromatographic details were excluded after full-text review
Study characteristics	17	Cite each included study and present its characteristics.	The 22 included studies provided data on simultaneous quantification of multiple BPs (Table 2) from which 11 studies had calculated Hazard Indexes (Table 4)
Risk of bias in studies	18	Present assessments of risk of bias for each included study.	NA; a formal assessment of risk of bias for each individual study was not performed as the review focuses on analytical methodology and descriptive exposure assessments
Results of individual studies	19	For all outcomes, present, for each study: (a) summary statistics for each group (where appropriate) and (b) an effect estimate and its precision (e.g. confidence/credible interval), ideally using structured tables or plots.	The results showed a clear shift toward LC-MS/MS and highlighted cases where the Hazard Index exceeded 1
Results of	20a	For each synthesis, briefly summarise the characteristics and risk of bias	The synthesis confirmed LC-MS/MS as the most robust technique (lines 312-314)

PRISMA 2020 Checklist

Section and Topic	Item #	Checklist item	Location where item is reported
syntheses		among contributing studies.	and revealed that combined BP intake poses significant health risks under new TDI levels (lines 438-440)
	20b	Present results of all statistical syntheses conducted. If meta-analysis was done, present for each the summary estimate and its precision (e.g. confidence/credible interval) and measures of statistical heterogeneity. If comparing groups, describe the direction of the effect.	NA; no meta-analysis or formal statistical syntheses were conducted.
	20c	Present results of all investigations of possible causes of heterogeneity among study results.	Heterogeneity was explored qualitatively by comparing results across different food matrices and chromatographic techniques in Tables 2 and 4.
	20d	Present results of all sensitivity analyses conducted to assess the robustness of the synthesized results.	Sensitivity was assessed by including only those studies that successfully validated methods for multiple BP analogues simultaneously (lines 227-228).
Reporting biases	21	Present assessments of risk of bias due to missing results (arising from reporting biases) for each synthesis assessed.	Not applicable as no meta-analysis or formal statistical assessment of reporting bias was conducted.
Certainty of evidence	22	Present assessments of certainty (or confidence) in the body of evidence for each outcome assessed.	Not applicable as formal certainty assessment was not performed.
**DISCUSSION**			
Discussion	23a	Provide a general interpretation of the results in the context of other evidence.	The results are interpreted in light of the new EU near-zero tolerance limits, emphasizing the need for continuous monitoring of analogues – this is well described in Discussion section (4.2 Cumulative Exposure and Regulatory Shifts)
	23b	Discuss any limitations of the evidence included in the review.	Limitations – lines 470-489
	23c	Discuss any limitations of the review processes used.	Limitations – lines 470-489
	23d	Discuss implications of the results for practice, policy, and future research.	Future research – lines 490-502
**OTHER INFORMATION**			
Registration and protocol	24a	Provide registration information for the review, including register name and registration number, or state that the review was not registered.	The review was not registered
	24b	Indicate where the review protocol can be accessed, or state that a protocol was not prepared.	A formal protocol was not prepared; the review followed the PRISMA 2020 guidelines as outlined in the methods section.
	24c	Describe and explain any amendments to information provided at registration or in the protocol.	NA
Support	25	Describe sources of financial or non-financial support for the review, and the role of the funders or sponsors in the review.	Funding – Line 534-535
Competing interests	26	Declare any competing interests of review authors.	No conflict of interest; line 540
Availability of data, code and other materials	27	Report which of the following are publicly available and where they can be found: template data collection forms; data extracted from included studies; data used for all analyses; analytic code; any other materials used in the review.	All data extracted and used for analysis are presented within the article's tables and the reference list (lines 598-709)

From: Page MJ, McKenzie JE, Bossuyt PM, Boutron I, Hoffmann TC, Mulrow CD, et al. The PRISMA 2020 statement: an updated guideline for reporting systematic reviews. BMJ 2021;372:n71. doi: 10.1136/bmj.n71. This work is licensed under CC BY 4.0. To view a copy of this license, visit https://creativecommons.org/licenses/by/4.0/
